# Highlight report: New insights in liver physiology: Canalicular bile flux is diffusion dominated

**DOI:** 10.17179/excli2020-2836

**Published:** 2020-08-31

**Authors:** Ahmed Ezzat Ahmed

**Affiliations:** 1 Department of Biology, College of Science, King Khalid University, 61413 Abha, Asir, Saudi Arabia

## Abstract

One of the central functions of the liver is excretion of bile into the intestine. Currently, bile excretion is explained by the osmotic model, according to which bile acids are excreted by hepatocytes into the bile canaliculi and since bile acids are osmotically active they draw water into the canalicular lumen. Bile canaliculi are closed at the central side. Therefore, bile was postulated to flow to the open side into the ducts. However, bile flow in canaliculi has never been measured because of the small canalicular diameter which does not allow analysis of flux by conventional methods. Recently, methods have been developed that allow flow analysis in bile canaliculi and ducts. Interestingly, no measurable directed flow was observed in the canaliculi. Instead, small molecules in bile canaliculi reached the larger bile ducts by diffusion. Only there measurable flow sets in. The pathophysiological implications of this novel observation are discussed.

## ⁯⁯⁯⁯⁯⁯

In the current issue of Hepatology a novel concept has been published how the liver transports bile through canaliculi and ducts to the intestine (Vartak et al., 2020[20]). Hepatocytes are known to excrete bile acids and xenobiotics into a 'canal system' that finally drains into the intestine (Godoy et al., 2013[9]). The most upstream part of this canal system, the bile canaliculi, are lined by the membrane of hepatocytes. With only 0.5-2 µm diameter, bile canaliculi represent very thin vessels. They are connected to interlobular bile ducts by a connecting pipe, the so-called Hering channel (Vartak et al., 2016[19]). In contrast to bile canaliculi, the bile ducts are lined by epithelial cells, the cholangiocytes. In the present study, the authors show that it is important to differentiate bile canaliculi and bile ducts as functionally distinct domains (Vartak et al., 2020[20]): transport of small molecules in the bile canaliculi is diffusion dominated and only in the ducts diffusion is augmented by flow due to water influx (Vartak et al., 2020[20]). According to this model, bile canaliculi can be compared to a lake with standing water that is connected to a river, the bile duct. When a compound is given into the lake, it will also reach the river - by diffusion - and will only then be carried away by flow. This new model (Vartak et al., 2020[20]) contradicts the prevailing osmotic model of bile flow that is present in medical text books since decades (Boyer, 2013[2]; Boyer and Bloomer, 1974[3]; Boyer et al., 1970[4]; Layden et al., 1978[14]; Sperber, 1959[18]; Wheeler and Ramos, 1960[21]; Wheeler et al., 1968[22]; Wood et al., 1977[23]). According to the osmotic model, bile acids are excreted by hepatocytes into the bile canaliculi. Bile acids are osmotically active and draw water into the canalicular lumen. Since bile canaliculi are closed at their pericentral end, bile should flow to the open side into the ducts. However, bile flow in canaliculi has never been measured. Because of the small canalicular diameter flow analysis cannot be accomplished by conventional methods. Vartak and colleagues now established a method that allows the quantification of flow and diffusion in bile canaliculi and ducts in intact livers of living mice (Vartak et al., 2020[20]). For this purpose, they used a photoactivatable compound, CMNB-fluorescein, which only upon UV irradiation releases fluorescein that then can be detected. Importantly, CMNB-fluorescein is excreted into bile canaliculi. Using an intravital method, Vartak and colleagues photoactivated CMNB-fluorescein in small tissue regions of intact livers, simultaneously imaging the fluorescence generated in the UV exposed region. The result was unexpected, since the fluorescent material photoactivated in a small region of the canalicular network, spread symmetrically into the surrounding canaliculi, which indicates diffusion rather than flow. In contrast, when photoactivation was performed in a blood vessel, the photoactivated material rapidly moved unidirectionally with the blood flow. A particularly convincing set of data was obtained, when the authors photoactivated CMNB-fluorescein within a Hering channel. As expected, fluorescent material moved downstream into the bile duct; however, unexpectedly also travelled retrograde, upstream into the canalicular network. This retrograde flux would not be possible in a flow dominated system. 

In their previous work, the authors studied liver physiology based on mathematical models (Hoehme et al., 2010[11]; Schliess et al., 2014[17]; Bartl et al., 2015[1]; Schenk et al., 2017[16]; Ghallab et al., 2016[7]) and elucidated the microarchitecture of the biliary tract by imaging and 3D reconstruction (Hammad et al., 2014[10]; Damle-Vartak et al., 2019[5]; Friebel et al., 2015[6]). However, later they began to focus on intravital imaging of physiological processes (Reif et al., 2017[15]; Ghallab et al., 2019[8]; Köppert et al., 2018[13]). The present study demonstrates the importance of analyzing physiological parameters in intact organs *in vivo*, because it may be misleading to exclusively rely on model simulations. 

The findings of Vartak and colleagues overturn long-standing assumptions about how the liver excretes bile into the duodenum. The seemingly subtle difference between flow and diffusion becomes relevant, when it comes to identification of adequate therapeutic strategies for liver diseases, such as non-alcoholic fatty liver disease (NAFLD). In some cholestatic liver diseases the bile canalicular network shows alterations, such as limited connectivity to the ducts (Vartak et al., 2016[19]; Jansen et al., 2017[12]). If the same volume of liquid would have to pass the compromised canaliculi by advection, this should result in a build-up of pressure, which could damage liver tissue. Therefore, drugs should be identified that reduce the assumed flow, which would also prevent the increase of damaging pressure. However, based on the present results (Vartak et al., 2020[20]), this flow-pressure concept should be questioned. It will be interesting to observe the further discussion about the correct model of bile flux and its pathophysiological as well as clinical consequences. 

## Conflict of interest

The author declares that he has no conflict of interest. 
